# Food Composition Tables in Southeast Asia: The Contribution of the SMILING Project

**DOI:** 10.1007/s10995-018-2528-8

**Published:** 2018-06-08

**Authors:** Paul Hulshof, Esmee Doets, Sok Seyha, Touch Bunthang, Manithong Vonglokham, Sengchanh Kounnavong, Umi Famida, Siti Muslimatun, Otte Santika, Sri Prihatini, Nazarina Nazarudin, Abas Jahari, Nipa Rojroongwasinkul, Uraiporn Chittchang, Le Bach Mai, Le Hong Dung, Tran Thi Lua, Verena Nowak, Lucy Elburg, Alida Melse-Boonstra, Inge Brouwer

**Affiliations:** 1 Division of Human Nutrition, Wageningen University, P.O. Box 17, 6700 AA Wageningen, The Netherlands; 2 Wageningen Food & Biobased Research, Wageningen University and Research Centre, Wageningen, The Netherlands; 3 Division of Food and Nutrition Security, Department of Fisheries Post-Harvest Technologies and Quality Control Fisheries Administration, Phnom Penh, Cambodia; 4 National Institute of Public Health in Vientiane, Vientiane, Lao People’s Democratic Republic; 5 Southeast Asian Ministers of Education Organization Regional Center for Food and Nutrition/SEAMEO-RECFON, Jakarta, Indonesia; 6 National Institute of Health Research and Development, Ministry of Health, Jakarta, Indonesia; 7 Mahidol University, Bangkok, Thailand; 8 National Institute of Nutrition, Hanoi, Vietnam; 9 Bioversity International, Maccarese, Italy

**Keywords:** Food composition, SMILING project, Nutrient data, Capacity building, Data quality

## Abstract

*Objectives* Food composition data are key for many nutrition related activities in research, planning and policy. Combatting micronutrient malnutrition among women and young children using sustainable food based approaches, as aimed at in the SMILING project, requires high quality food composition data.* Methods* In order to develop capacity and to align procedures for establishing, updating and assessing the quality of key nutrient data in the food composition tables in Southeast Asia, a detailed roadmap was developed to identify and propose steps for this. This included a training workshop to build capacity in the field of food composition data, and alignment of procedures for selecting foods and nutrients to be included for quality assessment, and update of country specific food composition tables. The SEA partners in the SMILING project finalised a country specific food composition table (FCT) with updated compositional data on selected foods and nutrients considered key for designing nutrient dense and optimal diets for the target groups.* Results* Between 140 and 175 foods were selected for inclusion in the country specific FCTs. Key-nutrients were: energy, protein, total fat, carbohydrates, iron, zinc, (pro-)-vitamin A, folate, calcium, vitamin D, vitamin B1, vitamin B2, vitamin B3, vitamin B6, vitamin B12 and vitamin C. A detailed quality assessment on 13 key-foods per nutrient was performed using international guidelines. Nutrient data for specific local food items were often unavailable and data on folate, vitamin B12 and vitamin B6 contents were mostly missing. For many foods, documentation was not available, thereby complicating an in-depth quality assessment. Despite these limitations, the SMILING project offered a unique opportunity to increase awareness of the importance of high quality well documented food composition data.* Conclusion for Practise* The self-reported data quality demonstrated that there is considerable room for improvement of the nutrient data quality in some countries. In addition, investment in sustainable capacity development and an urgent need to produce and document high quality data on the micronutrient composition of especially local foods is required.

## Significance

Direct causes of child and maternal undernutrition are related to inadequate dietary intake and disease state. Assessing the adequacy of diets require high quality food composition data of foods consumed by the population at risk.

## Background

Information on the composition of foods is fundamental for programmes monitoring adequacy of dietary intake, research linking diet to health and disease, planning and prescription, education and food security (Elmadfa and Meyer 2010). Inadequate food composition data may lead to a failure in understanding the relationship between nutrient intake and health, or result in inappropriate, inefficient interventions to improve micronutrient status (Burlingame 2003; Harrison 2004). This link can be illustrated by the UNICEF conceptual framework that captures the multifactorial causality of undernutrition (UNICEF 2013). Especially the interplay between inadequate dietary intake and disease state are considered immediate key factors determining maternal and child undernutrition. Adequate dietary intake can be reached by access to affordable, diverse, and sufficient nutrient rich foods. Combatting micronutrient malnutrition among women and young children using sustainable food based approaches, as aimed at in the Sustainable Micronutrient Interventions to ControL Deficiencies and Improve Nutritional status and General Health in Asia (SMILING) initiative (Berger 2013), requires high quality data on the nutrient composition of foods consumed by the target population and preferably comprise information on the content of constituents that influence the bioavailability of specific nutrients. The SMILING initiative was a transnational collaboration of research and implementation institutions in five countries in Southeast Asia, namely, Cambodia, Indonesia, Laos PDR, Thailand and Vietnam, with European partners, to support the application of state-of-the-art knowledge to alleviate malnutrition in Southeast Asia. These Southeast Asian countries represent a range of social and economic development, extent of malnutrition, and differ in their capacity and success in nutrition improvement efforts. Several nutrition indicators show that malnutrition among children and women in the reproductive age is still common in these countries: stunting in children under 5 years of age range from 16 to 44%, underweight from 9 to 26%, wasting from 6 to 13% and anaemia from 29 to 55%; anaemia in non-pregnant women range from 14 to 43% and female underweight (BMI < 18.5 kg/m^2^) from 7 to 28% (WHO 2017). In the SMILING project, country specific food composition data were required for mathematical modelling to design nutrient dense and optimal diets in terms of acceptability, availability and affordability that support nutrition policy and intervention actions (Ferguson et al. 2006). The OPTIFOOD linear programming model, requires foods from each country to be regrouped into similar food groups and nutritional composition to be presented in an identical electronic format with adequate food and component description, and reliable nutrient values. Food composition tables (FCT) in the Southeast Asian (SEA) countries involved in the SMILING consortium, were expected to differ in many aspects. At the start of the SMILING project no published national food composition table was available for Cambodia and Laos PDR, except for an unpublished excel file (Cambodia) containing compositional information for 531 food items and a food book for dietary assessment (Laos) containing information for 148 food items. Differences between the food composition tables were expected at several levels: (1) at the food level (e.g. number of foods included in the table/database, language, food description details, representativeness for what is eaten), (2) at the nutrient level (e.g. component coverage & identification, analytical methods and modes of expression used for the nutrients), (3) at the value level (e.g. nutrient content, value type, extent of missing values, nutrient data source & acquisition), and (4) at the format and data base management level (e.g. compilation process, software tools, data interchange readiness) (Deharveng et al. 1999). In addition, the database quality, overall level of documentation, and the level of expertise on food composition data compilation was expected to vary between the partners involved. In order to develop capacity and to align procedures for establishing, updating and assessing the quality of key nutrient data in the food composition tables in Southeast Asia, a detailed roadmap was developed to identify and propose steps for this.

## Methods

### Programme of Actions

A one day workshop (Montpellier, March; 2012) was conducted to identify and define the steps and roadmap for creating, updating and assessing the quality of country specific food composition tables and to meet consensus on the nutrients to be included. The selection of nutrients for inclusion were: energy, protein, carbohydrates, iron, zinc, vitamin A plus pro-vitamin A carotenoids, folate, calcium, vitamin D, vitamin B12, B2, B6, C, B1 and niacin. This selection was largely based on prevalent public health problems in SEA countries related to micronutrient intake. Anti-nutrients, such as phytate were not included in the final selection of components because finding reliable food composition values for anti-nutrients was considered difficult due to the lack of standard analytical methods and limited sources with information on these components. Based on nutrient density and on national consumption data, up to ~ 200 foods were selected for inclusion in each of the country specific food composition tables. In order to enable each SEA country to provide an updated high quality FCT that included the nutritionally relevant food items and nutrients, and to assess the quality of the food composition data with special emphasis on key foods and key nutrients, a 2 week training workshop was planned in April 2012.

### Training Workshop

A training workshop was organized by Wageningen University in collaboration with the National Institute of Nutrition (NIN) in Hanoi, Vietnam. The objectives of the training workshop were threefold: to build capacity in the field of food composition compilation for the SEA partners involved in SMILING; to align procedures for selecting foods for quality assessment and update of the country specific food composition tables; and to align procedures for data checking and quality assessment of selected foods and nutrients. The workshop was facilitated by experts from the National Institute of Nutrition, Hanoi, Vietnam; from FAO-INFOODS Rome, Italy; from Mahidol University, Bangkok, Thailand; and from the Division of Human Nutrition, Wageningen University (WU), The Netherlands. The workshop was organized for selected individuals from the five SEA countries responsible for their countries food composition database. Two persons from each SEA country were trained. During the training, lectures, dynamic group interactions, e-learning, assignments, and excursions were included to address the issues listed in Table 1.

**Table 1:** Issues covered in the food composition training workshop

-Prioritization of foods and nutrients for inclusion into FCT
-Sampling of foods for analysis
-(Literature) sources of food composition
-Choice of analytical methods
-Review of methods of analysis of proximate constituents and micro nutrients
-Principles of compiling and updating
-Laboratory data quality and ASEAN activities for ensuring data quality
-Consequences of errors in FCT for research applications
-Component identification
-Value documentation
-Evaluation of data quality
-Guidelines for data checking prior to release of food composition table
-Quality assessment exercises
-Consequences of errors in FCT for research applications

In addition the procedure for production/update and quality assessment of country specific food composition tables as proposed by WU was discussed and extensively practiced with both facilitators and participants of the workshop. As a result a consensus document was produced: “Guidelines for the selection of foods for data checking and quality assessment of national food composition tables in Cambodia, Indonesia, Laos PDR, Thailand and Vietnam” (Hulshof et al. 2012). These guidelines were built on pre-existing guidelines as designed within EuroFIR (Salvini et al. 2012), INFOODS (FAO/INFOODS 2012) and ASEANFOODS (Puwastien et al. 2015). The procedure comprised five steps: (1) selection of foods and completion of the ~ 200 selected food item list with nutrient data, (2) Data check on all nutrients of the food item list using FAO/INFOODS guidelines, (3) Selection of key foods for detailed quality assessment, (4) Detailed quality assessment on key foods and selected micronutrients based on EuroFIR/ASEANFOODS standards, (5) Regrouping of foods into food groups required for OPTIFOOD linear modelling program.

### Selection of Foods and Completion with Nutrient Data

The selection of single food items to be included in the FCT was based on consumption of the food by more than 10% of the population. Or, when eaten by less than 10% of the population, the nutrient density of each food should be ranked among the five highest nutrient densities. Starting point for the completion of the nutrient data table was the national food composition table, or the one that was commonly used if no national food composition database existed (e.g. for Laos and Cambodia the ASEANFOODS table). In case of missing nutrient data for the food items, first regional tables with adequate documented data sources were consulted (primary ASEANFOODS, Thailand and Vietnam FCT) and, second, non-regional tables/databases were consulted, especially the USDA nutrient database. FAO/INFOODS guidelines for checking food composition data were used in order to make sure that the nutrient value adopted from a different data source matched the food item for which information was sought for, that component identification checks were done and documented, and that proper modes of expression were used (FAO/INFOODS 2012). In addition, internal consistency checks on nutrients and energy were done following the same FAO/INFOODS guidelines, e.g. sum of proximate components between 95 and 105 g/100 g. Establishing and updating the food composition table was done in excel spreadsheet program using the FAO/INFOODS compilation tool (Charrondiere and Burlingame 2011).

### Selection of Key Foods for Detailed Quality Assessment

**Fig. 1:**
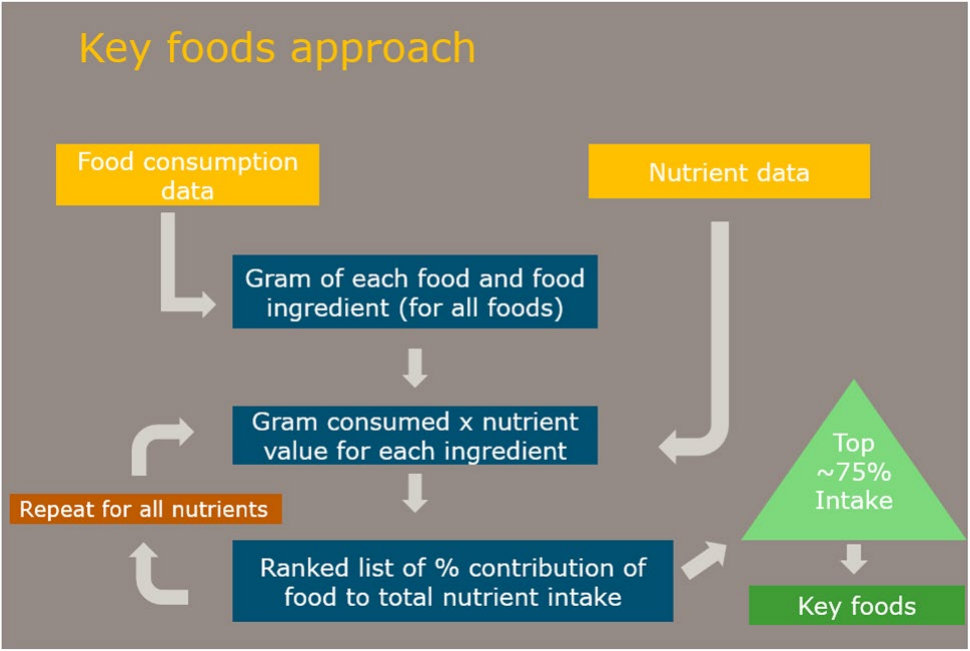
Key foods approach to select top ten foods that contribute most to intake of specific nutrient

Can we have confidence in the quality of the nutrient data if we want to use the food composition database for designing more optimized nutritious diets as aimed at in the SMILING project? A detailed quality assessment of all the nutrient values for the ~ 200 food item list ideally had to be done to answer this reliability question. However, considering the time constraints within the project, a fit for purpose assessment could only be done in a restricted number of food/nutrient combinations. The following micronutrients were included as they were of main public health concern: calcium, iron, zinc, vitamin A plus pro-vitamin A carotenoids, vitamin B1, B2, B12, C, folate and niacin. A key foods approach was used to select for each of the micronutrients the ~ 10 foods contributing most to intake in the respective country (Fig. 1) (Haytowitz et al. 2002). In addition, ~ 3 foods with the highest nutrient density, but underutilized, were also included. The unproven assumption was that the result of a detailed quality assessment in these foods would be a good approach to give insight in the overall quality of the database.

### Detailed Quality Assessment on Key Foods and Selected Micronutrients

**Fig. 2:**
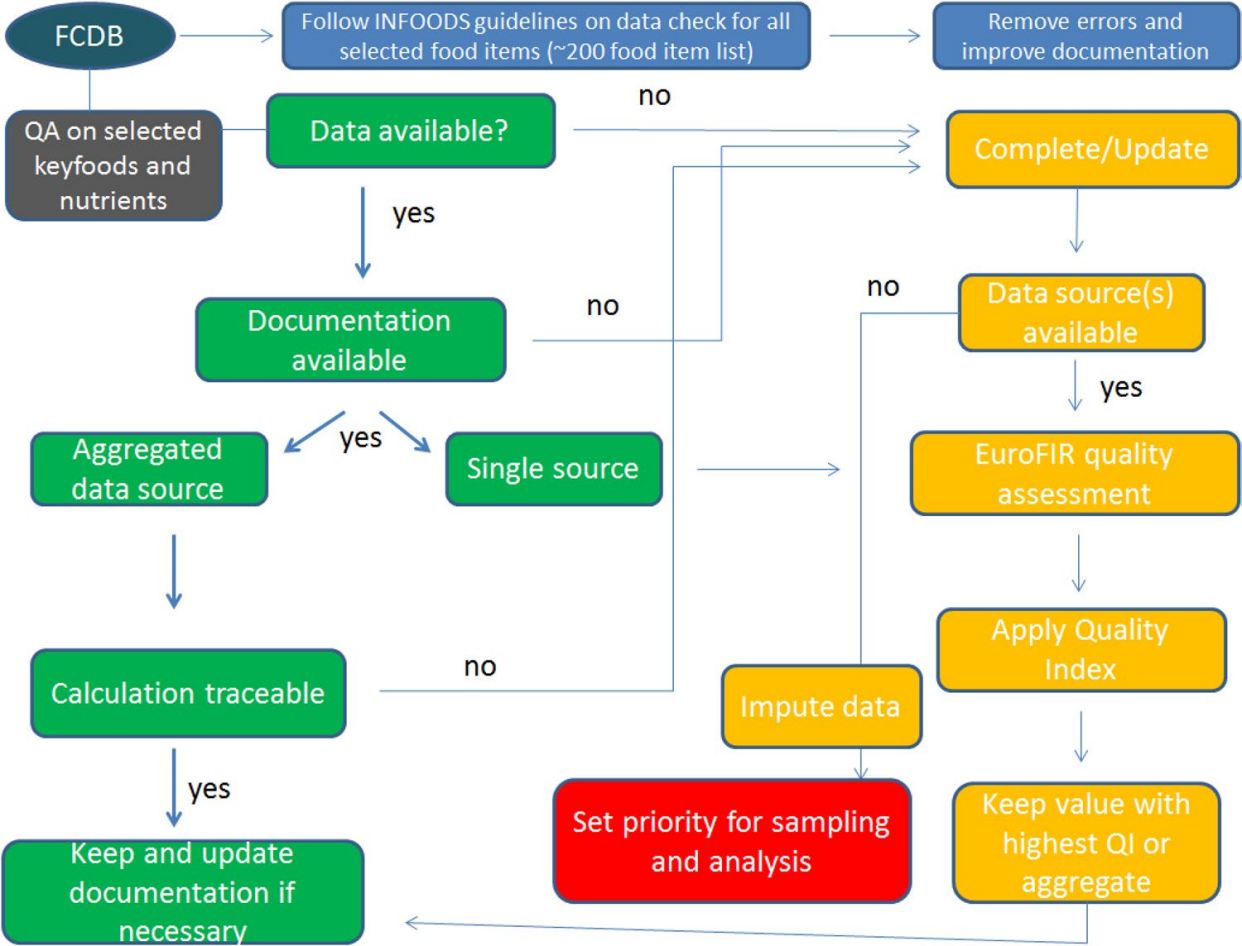
Flowchart for detailed quality assessment

The quality assessment approach on the key foods is summarized in a flowchart (Fig. 2). The basic philosophy of the flow chart is that it is not acceptable to have undocumented data in the food composition database, since undocumented data do not allow a quality assessment. In case no documentation for nutrient values existed, the values required updating from a documented data source. If no documented data source could be retrieved for the particular food-nutrient, data imputation was considered and priorities for sampling and analysis had to be set. Missing values in the databases were preferred to be as few as possible. Documented nutrient values from single sources were subjected to a detailed quality assessment. In case compositional data came from multiple independent documented data sources (aggregated data) with a traceable and acceptable calculation of the nutrient value based on the criteria of ASEANFOODS (Puwastien et al. 2015), the data were kept and not further submitted for a detailed quality assessment. This pragmatic approach seemed to be justified by face validity and was considered “best practise” to reduce the overall workload of the examiners.

EuroFIR guidelines were used for quality assessment of original documented data (Salvini et al. 2012), i.e. food composition data from journal articles, laboratory reports and similar as opposed to compiled food composition tables. These guidelines are based on the consensus that high quality nutrient data in a food composition database is determined by an adequate sampling plan, adequate food description, sufficient number of analytical samples, appropriate analytical methodology, and acceptable analytical performance (Holden et al. 2002; Southgate 2002). The application of these guidelines was extensively practised in the 2 weeks training course at NIN in Hanoi and partners were supposed to do a self-assessment and assign a quality index (QI) to the nutrient data, and to document the quality assessment using standardized formats (example in Table 2).

**Table 2:** Example of completed format for quality assessment of micro nutrient values in key foods (adapted from Salvini et al. 2012)

Nutrient	Iron			
INFOODS component tag name	FE			
Food name (English)	Rice, polished, steamed			
Food code (FC)	01015			
Source (reference) available? (Answer: yes or no)	Yes			
Full reference: Puwastein et al. (2015)				
Content (unit/matrix unit)	0.20 mg / 100 g			

	YES	NO	N/A	QI
**FOOD DESCRIPTION**				
*FOR ALL TYPES OF FOOD*				
Is the food group known?	x			
Was the food source of the food or of the main ingredient clearly provided?	x			
Was the part of plant or part of animal clearly indicated?			x	
If relevant, was the analysed portion described and is it stated explicitly if the food was analysed with or without the inedible part?	x			
If relevant, was the extent of heat treatment provided?	x			
If the food was cooked, were satisfactory cooking method details provided?		x		
Was relevant information on treatment applied provided?		x		
Was information on preservation method provided?			x	
If relevant, was information on packing medium provided?			x	
(If relevant) Was information about the origin of food provided?	x			
If relevant, was the month or season of production indicated?		x		
Was the moisture content of the sample measured and the result given?	x			
*FOR MANUFACTURED PREPACKED FOOD ONLY*				
Was generic name provided?			x	
Was commercial name provided?			x	
If relevant, was brand name provided?			x	
Was relevant information on consumer group/dietary use/label claim provided?			x	
Was recipe description provided?			x	
**Food Description QI: Y/(Y + N) × 5**				3
**COMPONENT IDENTIFICATION**				
Is the component described unambiguously?	x			
Is the unit unequivocal?	x			
Is the matrix unit unequivocal?	x			
**Component identification QI**				5
**SAMPLING PLAN**				
Was the sampling plan developed to represent consumption in the country where the study was conducted?		x		
Was the number of primary samples > 9?	x			
If relevant, were samples taken during more than one season of the year?		x		
If relevant, were samples taken from more than one geographical location?		x		
If relevant, were samples taken from the most important sales outlets (supermarket, local grocery, street market, restaurant, household…)?			x	
If relevant, was more than one brand (for manufactured pre-packed product) or more than one cultivar (for plant foods) or subspecies (for animal foods) sampled?	x			
**Sampling plan QI**				2
**NUMBER OF ANALYTICAL SAMPLES**				
Is the number of analytical samples 1?				
Is the number of analytical samples 2?	x			
Is the number of analytical samples 3?				
Is the number of analytical samples 4?				
Is the number of analytical samples ≥ 5?				
**Number of analytical samples QI**				2
**SAMPLE HANDLING**				
If relevant, were appropriate stabilization treatments applied (e.g. protection from heat/air/light/microbial activity)?		x		
Were the samples homogenized?	x			
**Sample handling QI**				3
**ANALYTICAL METHOD**				
Does the analytical method used in the source match the list of appropriate analytical methods given in the guidelines for analytical methods?	x			
Are the key method steps appropriate for the method described?	x			
**Analytical method QI**				5
**ANALYTICAL QUALITY CONTROL**				
Were analytical portion replicates tested?	x			
Was the laboratory accredited for this method or was the method validated by performance testing?	x			
If available, was an appropriate reference material or a standard reference material used?	x			
**Analytical quality control QI**				5
**Overall QI**				25
**Normalized QI: (overall QI–7)/(35–7) × 10**				6.4

### Regrouping of Foods

For the purpose of linear modelling, the food items in each of the completed and updated databases were regrouped into the following main food groups: legumes, nuts and seeds, meat, fish and eggs, starchy roots and other starchy plant products, vegetables, grain and grain products, fruits, dairy products, bakery and breakfast cereals, added fats, added sugars, composites, beverages, savoury snacks, sweetened snacks and desserts, special fortified products, human milk, and miscellaneous foods.

## Results

The steps taken as described above resulted in new or updated country specific food composition tables in an excel format containing food items consumed by more than 10% of the population and nutrient dense foods with compositional data for energy, water, ash, fat, protein, total dietary fibre, available carbohydrates, calcium, iron, zinc, vitamin A plus pro-vitamin A carotenoids, folate, vitamin D, vitamin B12, B2, B6, C, B1 and niacin. The number of food items included ranged from 90 for Cambodia, up to 174 for Indonesia. The food composition sources used for updating comprised (local) analytical reports, regional food composition databases (especially from ASEANFOODS, Vietnam, Thailand, Philippines and Malaysia), and non-regional sources (mainly USDA). In many—mainly local- foods, missing values particularly remained existent for vitamin D, vitamin B12, vitamin B6, niacin and folate. Table 3 shows the self-reported QI for selected nutrients on a scale from 0 to 10. For Cambodia the normalized QI ranged from 4.2 to 4.7; for Thailand from 7 to 8.2; for Vietnam from 6.4 to 6.7; and for Indonesia from 4.1 to 6.6. No QI was reported by Laos due to lack of documentation in all scoring categories (food description, component identification, sampling plan, number of samples, sample handling, analytical method and performance). For several nutrients (vitamin B6, B12, and folate) insufficient or no documentation of the scoring categories was at hand to allow an assessment. Lack of documentation on sampling plans was reported by Cambodia and Vietnam for all nutrients under consideration.

**Table 3:** Self-reported QI of selected nutrients for “top 10” foods that contribute to intake for specific nutrient

	Ca	Fe	Zn	B1	B2	B6	B12	Folate	Vitamin A	Vitamin C
Cambodia (3.4–5.1)	4.3	4.7	4.3	4.5	4.7	4.2	4.3	4.7	4.5	4.6
Thailand (5.7–9.2)	7.6	7	7.6	7.3	7.9	7.2	7.3	7.3	8.2	8.0
Vietnam (6.1–9.4)	6.6	6.4	6.4	6.5	6.5	n.a.	n.a.	n.a.	6.4	6.7
Laos (no QA)	n.a.	n.a.	n.a.	n.a.	n.a.	n.a.	n.a.	n.a.	n.a.	n.a.
Indonesia (2.6–8.2)	5.6	6.1	5.9	5.2	6.6	6.6	n.a.	n.a.	5.6	4.1

## Discussion

Nutritional deficiencies should preferably be combated with nutritious foods and in order to enable that, high quality data on the nutritional composition of foods are essential. Here we described the steps and approach that (1) contributed to capacity building in SEA countries for the creation of internationally recognized high quality food composition tables; (2) provided updated FCT for each SEA country for selected foods and nutrients that can serve as source for designing optimized diets using linear modelling; (3) assessed the data quality with emphasis on key foods and key nutrients of the updated FCT.

The limited time span of the project required prioritization and a clear roadmap. At the start of the project the major challenge faced was getting timely access to national food consumption data in order to be able to select foods consumed by more than 10% of the target population. As a consequence, a rapid dietary assessment had to be carried out first by one of the countries.

The completion of the national FCT with nutrient data taken from analytical reports, regional and international food composition sources sufficed for most nutrients but not for vitamin B6, B12, D and folate. For these nutrients compositional data for local foods were often not available. Of concern were errors that could arise when values from different data sources were mixed. To strengthen consciousness that the consulted data sources may use different definitions for nutrients, different modes of expression, different analytical procedures, and different descriptions for the food items, the FAO/INFOOD guidelines were used in the standardization process. These guidelines allowed checks on validity and internal consistency of the data, thus helping to solve inconsistencies. Although nutrient values were often not available for vitamin B6, B12, D, and folate in local foods, some of the data sources that were used to complete the data were quite old or copied from non-regional sources, thereby risking mismatch of foods and nutrients where the information was sought for. Since foods are biological material, nutrient values vary due to natural variation depending on soil and climate, plant and animal husbandry, storage, (home) processing, manufacturing and mixing of foods (Greenfield and Southgate 2003). In copying data from other data sources, the compiler should be aware of this variation and also of the uncertainties introduced during sampling, sample handling, and during chemical analysis and compilation.

The diverse origin of food composition data requires a systematic and documented approach to warrant good quality data. In this project, estimation of the data quality using EuroFIR guidelines was often hampered by the lack of documentation regarding the accomplishment of the nutrient values. The self-reported QI (Table 3), showed that the confidence that the user can have in the food composition data should in general be judged as “moderate”. Since these reports were based on self-assessments, there seems to be considerable room for improvement of the data quality in some countries, e.g. by reduction of random and systematic errors during the data generation and compilation process, and by improved documentation. Random and systematic errors become fixed once entered in a FCDB and may propagate to other FCDBs when data are copied. In measuring exposure from diet, as is the case in monitoring and surveillance in public health research, errors may lead to a biased estimate of the proportion of the population above or below the recommended dietary allowance, or lead to inappropriate, ineffective interventions to combat hidden hunger. In nutritional epidemiology, errors in FCDB may lead attenuated associations when studying the relationship between intake and health or disease (Willet 2012).

Guidelines for the assessment of the quality of existing food composition data are mostly limited to internal validity and consistency checks (Siebelink et al. 2015). The approach used in the SMILING project to estimate the fit for purpose of the FCT (can we have sufficient confidence in the data to use them for designing optimal diets?) was based on internal consistency/validity checks on the nutrient composition of foods eaten by more than 10% of the population and on underutilized nutrient dense foods by using the FAO/INFOODS guidelines. In addition a detailed assessment was done on a limited number of key food/key nutrient combinations assuming that such an approach would give a good insight in the overall quality of the database. In the presence of adequate documentation such an approach may be a fruitful method and give sufficient grip on the fit for purpose of a database. However, it would require independent assessment instead of self-assessment, and validation by (re)sampling and processing of foods, and submission of the foods for chemical analysis using state of the art analytical methodologies. The SMILING project could have benefitted from such an approach, but this was not compatible with other competing and demanding tasks in the project.

In conclusion: prioritization and characterization of the best strategies to translate knowledge into policy and action as aimed at in the SMILING initiative should have a sound base. Food composition tables are at the base of these activities. Strategies to ensure adequate dietary intake for the vulnerable population requires knowledge of the nutrient composition of foods. Food composition databases to support these strategies should comprise representative data of sound quality, comprehensive coverage of (local) foods and nutrients, clear food and component descriptions and documented data origins.

The SMILING project offered a unique opportunity to increase awareness of the importance of high quality well documented food composition data and provided updated FCT’s with a documented QI for feeding into the OPTIFOOD linear modelling program. The self-assigned QI demonstrated considerable room for improvement of the nutrient data quality in some countries. Lessons for the future and priority for research are twofold: first, a continuous update and investment in sustainable capacity remains crucial to secure maintenance and improvement of each countries FCT; second, there remains an urgent need to produce and document high quality data on the nutrient composition of especially local foods (notably folate, niacin, vitamin B6, B12, and vitamin D) and a plan to replace “old” data.
